# 5-Fluoro-1-(4-meth­oxy­benz­yl)indoline-2,3-dione

**DOI:** 10.1107/S1600536811023488

**Published:** 2011-06-30

**Authors:** Weiyao Wu, Huihui Lin, Chong-Qing Wan, Sheng-Li Cao

**Affiliations:** a Department of Chemistry, Capital Normal University, Beijing 100048, People’s Republic of China

## Abstract

In the title compound, C_16_H_12_FNO_3_, the dihedral angle between the benzene ring and the plane of the indole ring system is 71.60 (6)°. In the crystal, mol­ecules stack along the *b* axis through π–π inter­actions between the adjacent indole-2,3-dione units with a centroid–centroid distance of 3.649 (3) Å. Inter­molecular C—H⋯O=C and C—H⋯π inter­actions further stabilize the structure, forming a three-dimensional framework.

## Related literature

For background to the use of 5-fluoro­indoline-2,3-dione and its analogues as anti-tumour agents, see: Uddin *et al.* (2007 ▶); Penthala *et al.* (2010 ▶). For a related structure, see: Wu *et al.* (2011 ▶).
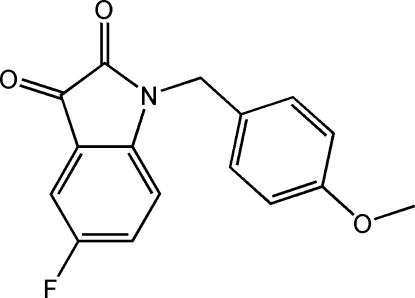


## Experimental

### 

#### Crystal data

C_16_H_12_FNO_3_
*M* *_r_* = 285.27
Orthorhombic, 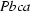

*a* = 17.779 (4) Å
*b* = 7.1575 (14) Å
*c* = 21.306 (4) Å
*V* = 2711.3 (9) Å^3^
*Z* = 8
Mo *K*α radiation
μ = 0.11 mm^−1^
*T* = 296 K
0.40 × 0.30 × 0.20 mm

#### Data collection

Bruker APEXII CCD area-detector diffractometer
Absorption correction: multi-scan (*SADABS*; Bruker, 2007 ▶) *T*_min_ = 0.641, *T*_max_ = 0.746
25325 measured reflections
3202 independent reflections
1604 reflections with *I* > 2σ(*I*)
*R*_int_ = 0.100

#### Refinement

*R*[*F* ^2^ > 2σ(*F* ^2^)] = 0.049
*wR*(*F* ^2^) = 0.136
*S* = 1.01
3202 reflections
190 parameters
H-atom parameters constrained
Δρ_max_ = 0.16 e Å^−3^
Δρ_min_ = −0.15 e Å^−3^

### 

Data collection: *APEX2* (Bruker, 2007 ▶); cell refinement: *APEX2* and *SAINT* (Bruker, 2007 ▶); data reduction: *SAINT*; program(s) used to solve structure: *SHELXS97* (Sheldrick, 2008 ▶); program(s) used to refine structure: *SHELXL97* (Sheldrick, 2008 ▶); molecular graphics: *SHELXTL* (Sheldrick, 2008 ▶); software used to prepare material for publication: *SHELXTL* and *PLATON* (Spek, 2009 ▶).

## Supplementary Material

Crystal structure: contains datablock(s) I, global. DOI: 10.1107/S1600536811023488/sj5156sup1.cif

Structure factors: contains datablock(s) I. DOI: 10.1107/S1600536811023488/sj5156Isup2.hkl

Supplementary material file. DOI: 10.1107/S1600536811023488/sj5156Isup3.cml

Additional supplementary materials:  crystallographic information; 3D view; checkCIF report

## Figures and Tables

**Table 1:** Hydrogen-bond geometry (Å, °) *Cg*1 is the centroid of the C1–C6 benzene ring.

*D*—H⋯*A*	*D*—H	H⋯*A*	*D*⋯*A*	*D*—H⋯*A*
C15—H15*A*⋯*Cg*1^i^	0.93	3.03	3.812 (2)	142
C14—H14*A*⋯O2^ii^	0.93	2.49	3.345 (2)	153

Symmetry codes: (i) 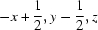; (ii) 
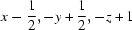
.

**Fig. 1.**
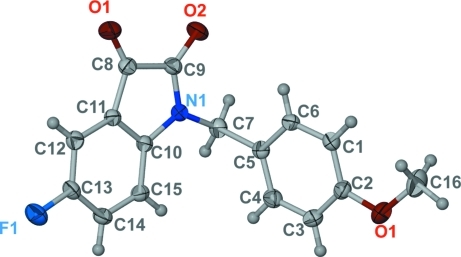
The title molecule with the atomic numbering scheme. The displacement ellipsoids are shown at the 30% probability level, while the hydrogen atoms are shown as spheres of arbitrary radius.

**Fig. 2.**
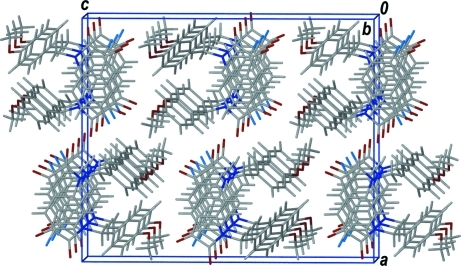
View down the b axis of the crystal packing of the title compound.

## supplementary crystallographic information

### Comment

The derivatives of 5-fluoroindoline-2,3-dione and its analogues are widely used as anti-tumor compounds (Uddin *et al.*,  2007; Penthala *et al.*, 2010). Herein, we report the crystal structure of one new derivative of 5-fluoroindoline-2,3-dione.

In the title compound C_16_H_12_FNO_3_, the indoline moiety links to the 4-methoxybenzene through methylene group with a C5-C7(methylene)-N1 angle of 113.29 (2)° (Fig. 1). The benzene ring and the plane of the indole-2,3-dione exhibit a dihedral angle of 71.60 (6)°. The C5-C7(methylene)-N1 angle and the dihedral angle are comparable to these in the chloro-substituted compound, 5-chloro-1- (4-methoxybenzyl)indoline-2,3-dione, where the corresponding values are 113.86 (2)° and 88.44 (8)° (Wu *et al.* 2011). Molecules stack along the *b* axis through π–π stacking interactions between adjacent indole-2,3-dione units with a Cg···Cg distance of 3.649 (3)Å and C15-H15A···Cg contacts, Table 1, form a chain  structure, as shown in Fig. 2. The almost parallel chains are further interconnected through C14-H14A···O2═C9 interactions, Table 1, generating a three-dimensional framework, Fig. 2.

### Experimental

To an ice-bath cooled solution of 5-fluoroindoline-2,3-dione (0.33 g, 2 mmol) in N,N-dimethylformamide (20 ml) was added potassium carbonate (0.33 g, 2.4 mmol) and potassium iodide (0.07 g, 0.4 mmol) followed by 4-methoxybenzyl chloride (0.32 ml, 2.2 mmol). The reaction mixture was stirred at 110 °C for 3 h. After cooling to room temperature, the reaction mixture was poured into ice water (80 ml). The resulting precipitate was filtered and subsequently purified by column chromatography on silica gel with dichloromethane as an eluent to give the title compound (Rf = 0.81, dichloromethane; m.p. 138-139 °C; yield 78%). Yellow crystals of the title compound were obtained by slow evaporation from the solution of dichloromethane/ethanol 8:2 (v/v) at room temperature after a week.

### Refinement

All the H atoms were discernible in the difference electron density maps. Nevertheless, the hydrogen atoms were placed into idealized positions and allowed to ride on the carrier atoms, with C—H = 0.93 and 0.97 Å for aryl and methylene hydrogens, respectively. *U*_iso_(H) = 1.2*U*_eq_(C)_aryl_/_methylene_.

### Crystal data

**Table:**

C_16_H_12_FNO_3_	*Z* = 8
*M**_r_* = 285.27	*F*(000) = 1184
Orthorhombic, *P**b**c**a*	*D*_x_ = 1.398 Mg m^−^^3^
Hall symbol: -P 2ac 2ab	Mo *K*α radiation, λ = 0.71073 Å
*a* = 17.779 (4) Å	µ = 0.11 mm^−^^1^
*b* = 7.1575 (14) Å	*T* = 296 K
*c* = 21.306 (4) Å	Block, yellow
*V* = 2711.3 (9) Å^3^	0.40 × 0.30 × 0.20 mm

### Data collection

**Table:**

Bruker APEXII CCD area-detector diffractometer	3202 independent reflections
Radiation source: fine-focus sealed tube	1604 reflections with *I* > 2σ(*I*)
graphite	*R*_int_ = 0.100
ω scans	θ_max_ = 27.9°, θ_min_ = 6.4°
Absorption correction: multi-scan (*SADABS*; Bruker, 2007)	*h* = −23→23
*T*_min_ = 0.641, *T*_max_ = 0.746	*k* = −9→9
25325 measured reflections	*l* = −28→27

### Refinement

**Table:**

Refinement on *F*^2^	Primary atom site location: structure-invariant direct methods
Least-squares matrix: full	Secondary atom site location: difference Fourier map
*R*[*F*^2^ > 2σ(*F*^2^)] = 0.049	Hydrogen site location: inferred from neighbouring sites
*wR*(*F*^2^) = 0.136	H-atom parameters constrained
*S* = 1.01	*w* = 1/[σ^2^(*F*_o_^2^) + (0.0557*P*)^2^ + 0.1083*P*] where *P* = (*F*_o_^2^ + 2*F*_c_^2^)/3
3202 reflections	(Δ/σ)_max_ < 0.001
190 parameters	Δρ_max_ = 0.16 e Å^−^^3^
0 restraints	Δρ_min_ = −0.15 e Å^−^^3^

### Special details

**Table:**

Geometry. All esds (except the esd in the dihedral angle between two l.s. planes) are estimated using the full covariance matrix. The cell esds are taken into account individually in the estimation of esds in distances, angles and torsion angles; correlations between esds in cell parameters are only used when they are defined by crystal symmetry. An approximate (isotropic) treatment of cell esds is used for estimating esds involving l.s. planes.
Refinement. Refinement of F^2^ against ALL reflections. The weighted R-factor wR and goodness of fit S are based on F^2^, conventional R-factors R are based on F, with F set to zero for negative F^2^. The threshold expression of F^2^ > 2sigma(F^2^) is used only for calculating R-factors(gt) etc. and is not relevant to the choice of reflections for refinement. R-factors based on F^2^ are statistically about twice as large as those based on F, and R- factors based on ALL data will be even larger.

### Fractional atomic coordinates and isotropic or equivalent isotropic displacement parameters (Å^2^)

**Table:**

	*x*	*y*	*z*	*U*_iso_*/*U*_eq_	
F1	0.08487 (7)	0.2819 (2)	0.39404 (6)	0.0874 (4)	
O1	0.38817 (8)	0.2815 (2)	0.34962 (7)	0.0788 (5)	
O2	0.47658 (8)	0.2065 (2)	0.46416 (7)	0.0738 (5)	
O3	0.36524 (10)	0.7189 (2)	0.74362 (7)	0.0833 (5)	
N1	0.35821 (8)	0.1683 (2)	0.50669 (7)	0.0508 (4)	
C1	0.42398 (10)	0.5766 (3)	0.65265 (9)	0.0569 (5)	
H1A	0.4579	0.6745	0.6482	0.068*	
C2	0.37197 (11)	0.5783 (3)	0.70053 (9)	0.0579 (5)	
C3	0.32168 (12)	0.4322 (3)	0.70640 (9)	0.0668 (6)	
H3A	0.2866	0.4325	0.7388	0.080*	
C4	0.32346 (12)	0.2861 (3)	0.66446 (10)	0.0630 (6)	
H4A	0.2890	0.1892	0.6687	0.076*	
C5	0.37553 (11)	0.2802 (3)	0.61599 (8)	0.0502 (5)	
C6	0.42526 (10)	0.4275 (3)	0.61115 (9)	0.0542 (5)	
H6A	0.4607	0.4268	0.5791	0.065*	
C7	0.37914 (11)	0.1181 (3)	0.57101 (9)	0.0576 (5)	
H7A	0.4299	0.0683	0.5709	0.069*	
H7B	0.3458	0.0202	0.5857	0.069*	
C8	0.36179 (12)	0.2478 (3)	0.40047 (10)	0.0543 (5)	
C9	0.40834 (11)	0.2057 (3)	0.45996 (9)	0.0545 (5)	
C10	0.28347 (10)	0.1884 (2)	0.48441 (9)	0.0451 (4)	
C11	0.28318 (10)	0.2373 (2)	0.42095 (9)	0.0472 (5)	
C12	0.21641 (11)	0.2671 (3)	0.38876 (9)	0.0552 (5)	
H12A	0.2157	0.2987	0.3464	0.066*	
C13	0.15144 (11)	0.2476 (3)	0.42286 (10)	0.0570 (5)	
C14	0.15047 (11)	0.1975 (3)	0.48525 (10)	0.0569 (5)	
H14A	0.1048	0.1840	0.5060	0.068*	
C15	0.21751 (10)	0.1671 (3)	0.51749 (9)	0.0522 (5)	
H15A	0.2177	0.1337	0.5597	0.063*	
C16	0.42269 (18)	0.8594 (4)	0.74439 (13)	0.0992 (9)	
H16A	0.4116	0.9495	0.7764	0.149*	
H16B	0.4245	0.9205	0.7043	0.149*	
H16C	0.4704	0.8021	0.7529	0.149*	

### Atomic displacement parameters (Å^2^)

**Table:**

	*U*^11^	*U*^22^	*U*^33^	*U*^12^	*U*^13^	*U*^23^
F1	0.0514 (7)	0.1155 (11)	0.0953 (10)	0.0063 (7)	−0.0130 (6)	0.0019 (8)
O1	0.0704 (10)	0.1040 (13)	0.0619 (10)	−0.0115 (8)	0.0144 (8)	0.0097 (8)
O2	0.0460 (9)	0.0904 (11)	0.0851 (11)	−0.0052 (7)	0.0015 (7)	0.0017 (8)
O3	0.0923 (12)	0.0894 (12)	0.0682 (10)	−0.0015 (9)	0.0081 (8)	−0.0262 (9)
N1	0.0480 (9)	0.0532 (9)	0.0510 (9)	−0.0020 (7)	−0.0001 (7)	−0.0018 (7)
C1	0.0512 (11)	0.0608 (13)	0.0587 (12)	−0.0066 (9)	−0.0012 (9)	−0.0035 (10)
C2	0.0604 (12)	0.0652 (13)	0.0480 (11)	0.0062 (11)	−0.0031 (10)	−0.0060 (10)
C3	0.0643 (13)	0.0856 (17)	0.0504 (12)	−0.0053 (12)	0.0107 (10)	−0.0008 (11)
C4	0.0614 (13)	0.0679 (14)	0.0597 (13)	−0.0122 (10)	0.0016 (10)	0.0056 (11)
C5	0.0499 (11)	0.0530 (12)	0.0479 (11)	0.0043 (9)	−0.0050 (9)	0.0054 (9)
C6	0.0448 (11)	0.0656 (13)	0.0520 (11)	0.0007 (10)	0.0024 (8)	−0.0009 (10)
C7	0.0609 (12)	0.0558 (12)	0.0560 (12)	0.0012 (9)	−0.0057 (10)	0.0028 (10)
C8	0.0558 (12)	0.0522 (12)	0.0549 (12)	−0.0070 (9)	0.0042 (10)	0.0004 (9)
C9	0.0483 (12)	0.0524 (12)	0.0628 (13)	−0.0044 (9)	0.0039 (10)	−0.0036 (9)
C10	0.0448 (10)	0.0388 (10)	0.0516 (11)	−0.0020 (8)	0.0028 (8)	−0.0063 (8)
C11	0.0472 (11)	0.0432 (10)	0.0513 (11)	−0.0031 (8)	0.0022 (8)	−0.0030 (8)
C12	0.0584 (13)	0.0533 (12)	0.0539 (12)	−0.0027 (9)	−0.0025 (9)	0.0002 (9)
C13	0.0448 (11)	0.0568 (12)	0.0694 (14)	−0.0004 (9)	−0.0068 (10)	−0.0036 (10)
C14	0.0466 (11)	0.0541 (12)	0.0699 (14)	−0.0051 (9)	0.0086 (9)	−0.0061 (10)
C15	0.0542 (12)	0.0488 (11)	0.0536 (11)	−0.0029 (9)	0.0079 (9)	−0.0027 (9)
C16	0.138 (3)	0.0752 (17)	0.0848 (18)	−0.0146 (18)	−0.0027 (16)	−0.0240 (14)

### Geometric parameters (Å, °)

**Table:**

F1—C13	1.356 (2)	C6—H6A	0.9300
O1—C8	1.205 (2)	C7—H7A	0.9700
O2—C9	1.217 (2)	C7—H7B	0.9700
O3—C2	1.368 (2)	C8—C11	1.466 (3)
O3—C16	1.433 (3)	C8—C9	1.544 (3)
N1—C9	1.363 (2)	C10—C15	1.377 (2)
N1—C10	1.418 (2)	C10—C11	1.396 (3)
N1—C7	1.465 (2)	C11—C12	1.388 (3)
C1—C2	1.377 (3)	C12—C13	1.372 (3)
C1—C6	1.386 (3)	C12—H12A	0.9300
C1—H1A	0.9300	C13—C14	1.377 (3)
C2—C3	1.382 (3)	C14—C15	1.393 (3)
C3—C4	1.375 (3)	C14—H14A	0.9300
C3—H3A	0.9300	C15—H15A	0.9300
C4—C5	1.388 (3)	C16—H16A	0.9600
C4—H4A	0.9300	C16—H16B	0.9600
C5—C6	1.380 (3)	C16—H16C	0.9600
C5—C7	1.507 (3)		
			
C2—O3—C16	117.50 (18)	O1—C8—C9	124.66 (19)
C9—N1—C10	110.37 (15)	C11—C8—C9	104.88 (16)
C9—N1—C7	124.44 (16)	O2—C9—N1	126.83 (19)
C10—N1—C7	125.18 (15)	O2—C9—C8	126.46 (18)
C2—C1—C6	119.38 (18)	N1—C9—C8	106.71 (16)
C2—C1—H1A	120.3	C15—C10—C11	121.35 (17)
C6—C1—H1A	120.3	C15—C10—N1	127.97 (17)
O3—C2—C1	124.24 (19)	C11—C10—N1	110.67 (15)
O3—C2—C3	116.10 (18)	C12—C11—C10	121.35 (16)
C1—C2—C3	119.66 (18)	C12—C11—C8	131.34 (17)
C4—C3—C2	120.11 (19)	C10—C11—C8	107.31 (16)
C4—C3—H3A	119.9	C13—C12—C11	116.29 (18)
C2—C3—H3A	119.9	C13—C12—H12A	121.9
C3—C4—C5	121.47 (19)	C11—C12—H12A	121.9
C3—C4—H4A	119.3	F1—C13—C12	118.47 (19)
C5—C4—H4A	119.3	F1—C13—C14	118.25 (18)
C6—C5—C4	117.37 (18)	C12—C13—C14	123.27 (18)
C6—C5—C7	120.92 (17)	C13—C14—C15	120.39 (18)
C4—C5—C7	121.70 (18)	C13—C14—H14A	119.8
C5—C6—C1	122.01 (17)	C15—C14—H14A	119.8
C5—C6—H6A	119.0	C10—C15—C14	117.34 (18)
C1—C6—H6A	119.0	C10—C15—H15A	121.3
N1—C7—C5	113.29 (15)	C14—C15—H15A	121.3
N1—C7—H7A	108.9	O3—C16—H16A	109.5
C5—C7—H7A	108.9	O3—C16—H16B	109.5
N1—C7—H7B	108.9	H16A—C16—H16B	109.5
C5—C7—H7B	108.9	O3—C16—H16C	109.5
H7A—C7—H7B	107.7	H16A—C16—H16C	109.5
O1—C8—C11	130.47 (19)	H16B—C16—H16C	109.5

### Hydrogen-bond geometry (Å, °)

**Table:**

Cg1 is the centroid of the C1–C6 benzene ring.

**Table:**

*D*—H···*A*	*D*—H	H···*A*	*D*···*A*	*D*—H···*A*
C15—H15A···Cg1^i^	0.93	3.03	3.812 (2)	142
C14—H14A···O2^ii^	0.93	2.49	3.345 (2)	153

Symmetry codes: (i) −*x*+1/2, *y*−1/2, *z*; (ii) *x*−1/2, −*y*+1/2, −*z*+1.
